# Insights into shell deposition in the Antarctic bivalve *Laternula elliptica*: gene discovery in the mantle transcriptome using 454 pyrosequencing

**DOI:** 10.1186/1471-2164-11-362

**Published:** 2010-06-08

**Authors:** Melody S Clark, Michael AS Thorne, Florbela A Vieira, João CR Cardoso, Deborah M Power, Lloyd S Peck

**Affiliations:** 1 British Antarctic Survey, Natural Environment Research Council, High Cross, Madingley Road, Cambridge, CB3 0ET, UK; 2 Center of Marine Sciences, Universidade do Algarve, Campus de Gambelas,8005-139 Faro, Portugal

## Abstract

**Background:**

The Antarctic clam, *Laternula elliptica*, is an infaunal stenothermal bivalve mollusc with a circumpolar distribution. It plays a significant role in bentho-pelagic coupling and hence has been proposed as a sentinel species for climate change monitoring. Previous studies have shown that this mollusc displays a high level of plasticity with regard to shell deposition and damage repair against a background of genetic homogeneity. The Southern Ocean has amongst the lowest present-day CaCO_3 _saturation rate of any ocean region, and is predicted to be among the first to become undersaturated under current ocean acidification scenarios. Hence, this species presents as an ideal candidate for studies into the processes of calcium regulation and shell deposition in our changing ocean environments.

**Results:**

454 sequencing of *L. elliptica *mantle tissue generated 18,290 contigs with an average size of 535 bp (ranging between 142 bp-5.591 kb). BLAST sequence similarity searching assigned putative function to 17% of the data set, with a significant proportion of these transcripts being involved in binding and potentially of a secretory nature, as defined by GO molecular function and biological process classifications. These results indicated that the mantle is a transcriptionally active tissue which is actively proliferating. All transcripts were screened against an in-house database of genes shown to be involved in extracellular matrix formation and calcium homeostasis in metazoans. Putative identifications were made for a number of classical shell deposition genes, such as tyrosinase, carbonic anhydrase and metalloprotease 1, along with novel members of the family 2 G-Protein Coupled Receptors (GPCRs). A membrane transport protein (SEC61) was also characterised and this demonstrated the utility of the clam sequence data as a resource for examining cold adapted amino acid substitutions. The sequence data contained 46,235 microsatellites and 13,084 Single Nucleotide Polymorphisms(SNPs/INDELS), providing a resource for population and also gene function studies.

**Conclusions:**

This is the first 454 data from an Antarctic marine invertebrate. Sequencing of mantle tissue from this non-model species has considerably increased resources for the investigation of the processes of shell deposition and repair in molluscs in a changing environment. A number of promising candidate genes were identified for functional analyses, which will be the subject of further investigation in this species and also used in model-hopping experiments in more tractable and economically important model aquaculture species, such as *Crassostrea gigas *and *Mytilus edulis*.

## Background

Laternulids are infaunal bivalve molluscs, which morphologically resemble the soft-shelled clam *Mya arenaria*, the major ingredient of clam chowder. In spite of a widespread latitudinal distribution ranging from the tropics, through temperate Australasia to Antarctica [1], research on this genus is dominated by work on the Antarctic species (*Laternula elliptica*). This clam has been studied for a number of years and is one of the best characterised Antarctic marine invertebrates. Studies initially focused on its ecology [2], and general physiology: reproduction [3,4], development [5,6], growth [7,8] and seasonal energetics [9,10]. However more recent research has focused on the longevity of this species in relation to reactive oxygen species production, antioxidant defences and cellular ageing [11], as this species often lives 25 years or more [12]. It has also been the subject of significant investigation of its thermal tolerance and the expected impact of climate change [13-15].

Antarctic marine invertebrates are stenothermal [14] and L. *elliptica *is one of the more sensitive species [13-16]. These animals suffer significant mortalities at 4-5°C, but lose essential biological functions, such as the ability to bury in sediment, much earlier, at only 1-2°C over current summer maximum sea water temperatures [13-15]. This thermal response is viewed against predictions that globally oceanic sea surface temperatures are predicted to rise on average by 2°C over the next 100 years [17,18]. However, regional differences are apparent and climate change along the Antarctic Peninsula has been particularly rapid with a temperature increase in the surface layers of the Bellingshausen Sea of 1°C in 50 years [19]. The predictions of the effect of these thermal changes on Antarctic marine biodiversity are complex [20] and further complicated by reductions in ocean pH.

Antarctic species, in general, have been proposed as excellent candidates for the development of climate change molecular biomarkers [21], whilst *L. elliptica *in particular has strong support as a sentinel species [22]. It has a circumpolar distribution and is highly abundant [23]. It is the largest individual mollusc in terms of live weight [7] and one of the highest in terms of total ecological biomass [24]. Being an infaunal filter-feeder, it also plays a significant role in benthopelagic coupling [25,26] and therefore is a keystone species of the Antarctic marine ecosystem.

In the 250 years since the onset of the industrial revolution, ocean pH has fallen from an average of 8.16 to 8.05 and is predicted to decrease by a further 0.3-0.4 pH units by the end of this century. These predicted changes in ocean pH are greater, and far more rapid, than any experienced in the past 300 million years [27-30]. The Southern Ocean will be particularly affected as it has amongst the lowest present-day CaCO_3 _saturation state of any ocean region, and will therefore be among the first to become undersaturated [31]. The ability of marine organisms to adapt to this unprecedented environmental modification of increased temperature and reduced pH is largely unknown, with particular concern expressed over calcifying animals such as echinoderms and molluscs [27].

*Laternula *species are conservative in shell form and habitat suggesting they share morphological constraints at different latitudes [32]. Recent data suggests however, that at least in the Southern Ocean, *L. elliptica *exhibits a high degree of plasticity in the thickness of shell deposition. This physical characteristic varies markedly between locations along the Antarctic Peninsula. Specimens at Rothera base, Adelaide Island (67° 4' 07" S, 68° 07' 30" W) have shells at least 2-3× thicker than those at Jubany base King George Island (62.23°S, 58.67°W) (Harper, pers comm). The Rothera animals also have damage repair rates 5-12× higher than Jubany animals, possibly due to increased frequency of iceberg impact (Harper, pers comm). This plasticity in shell thickness is superimposed against a homogeneous population structure across all sites (Hoffman, pers comm). Such plasticity in shell deposition has also been observed in the Antarctic limpet, *Nacella concinna *[33].

Shells of *L. elliptica *were recently subjected to an end of century scenario pH 7.4 trial and showed extensive damage over 56 days [34]. However, these were isolated shells and such studies do not take into account the ability of the animal to ameliorate shell loss via increased deposition or the effects of having an infaunal life habit. To understand the dynamics of shell turnover and the response of this process to both biotic and abiotic factors a better understanding of the molecular basis and regulation of shell formation is required. The studies which do exist have revealed that the molluscan shell is composed largely of calcium carbonate and organic macromolecules which are secreted by the mantle [35-40]. Hence, whole animal studies are essential not only to understand the shell deposition process in relation to altered temperature and pH, but also the more subtle effects of altered environmental conditions on calcium regulation in cellular processes and the energetic trade-offs of responses to climate change.

The aragonitic shell of *L.elliptica *comprises two layers: a very thin prismatic layer on the outside covering layers of sheet nacre on the inner side (with sheet nacre on both sides of the pallial myostracum); and a periostracum of around 10 μm surrounds the outer shell where it has not been removed by abrasion [[41,42]; Harper, pers comm.]. The mantle secretes the shell and forms a large thin sheet of tissue between the shell and the internal organs and extends beyond the rest of the body, so that part of the edge is exposed to external conditions [42]. It thus serves two purposes, secretion of the shell matrix and also protection from the external environment, either via sealing the edge of the shell or from damage to the shell itself (for example, after being impacted by an iceberg (Peck, pers comm)). The edge of the mantle comprises three folds, of which, only the outer fold is involved in laying down the actual shell material. The periostracum emerges from the groove between the middle and outer folds and bends back to cover the shell. The primary role of the perisotracum is believed to be shell secretion and maintenance, but there are other roles such as protection from the external environment, infestation and predatory borers [42]. The mantle tissue for this analysis was taken as a cross section of all mantle folds and included periostracum material.

**Figure 1:**
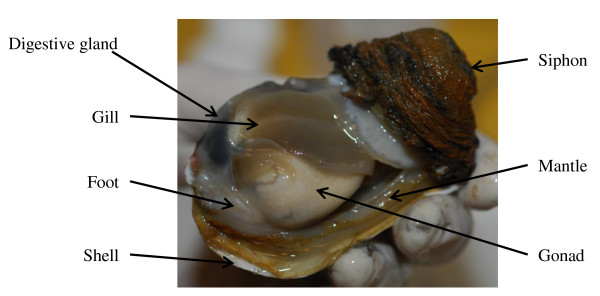
**Annotated longitudinal dissection of *L. elliptica *with one of the shells removed. Photograph copyright permission obtained from Erwan Amice**.

So far molecular work in *L. elliptica *has been limited to candidate genes concerned with understanding thermal tolerance via antioxidants and heat shock proteins [[43-45], Truebano et al, submitted]. The aim of this study is to develop molecular resources for this species. We focus on the mantle, as it is the main shell secreting organ (Figure 1) and we are particularly interested in the processes of calcification and comparative ossification [46-48]. 454 pyrosequencing technologies enable the rapid generation of transcriptomes for non-model species [49]. This approach is exploited in the present study, the results of which will be the basis of future studies of calcium regulation in *L. elliptica *in relation to environmental change. Here we describe the transcriptome of the mantle tissue of *L. elliptica*, focussing on the datamining of genes involved in calcium regulation and shell deposition. This represents the first publicly available 454 data for an Antarctic marine invertebrate and provides an important comparative resource for such studies in more eurythermal temperate mollusc species.

## Results and Discussion

The non-normalised laternula libraries were subjected to a full 454 run that yielded 1,034,155 reads totalling 381,838,384 bases with an average read length per trancript of 369 bases. After cleaning the data and removing small reads, 778,629 reads with an average size of 304 bp were entered into Newbler for assembly. These assembled into the 18,290 contigs (264,289 reads) which were used for further analysis. Because the aim of this project was to identify and characterise specific genes for future analyses, in particular the GPCRs and several gene families, such as collagen and bone morphogenic proteins (Additional files: Tables S1 and S2) there was a requirement for longer sequences of good quality which would enable us to distinguish between gene family members. Hence the descriptive analysis presented here utilised only the contigs produced by the assembly. Whilst the singletons potentially contain useful lowly expressed sequences, they also contain a substantial proportion of artefacts derived from cDNA synthesis, sequencing and contamination [50]. PCR and re-sequencing of singletons is essential in order to verify the gene products [50]. The contigs ranged in size from 142 bp to 5591 bp, with an average size of 535 bp. 42 contigs were greater than 3 kb and 69 contigs comprised more than 300 reads, with the largest contig of 5591 bp comprising the most reads with 1000 sequences (Table 1). Self BLAST of this dataset produced only 281 matches with a value of e^-100^, indicating a low level (<1.5%) of redundancy in the assembly of the reads. The contigs contained 46,235 microsatellites, of which 1,608 comprised over 7 repeat units (Additional File Table S3). There were 13,084 SNPs/INDELS present in 2,475 contigs designated as high confidence by the Newbler program (Additional file Table S4), although a further circa 25,000 SNPs were identified at lower confidence level as defined by Newbler [51]. These figures for microsatellite and SNP/INDEL detection are at a similar relative level to those identified in the transcriptome of another non-model species, the flesh fly (*Sarcophaga crassipalpis*) [52]. Given that *L. elliptica *is a wild-caught species and the *S. crassipalpis *material came from a long standing inbred laboratory stock, higher levels of these genetic variants would be expected in the clam. However, analysis in this species was restricted to contigs only, potentially reducing the dataset.

**Table 1:** Most commonly expressed sequences with associated BLAST matches.

Contig ID	Length (bp)	No of reads	Description	Species	Common name	E-value
00447	5591	1000	Map kinase interacting serine threonine protein kinase.	Aplysia californica	California sea hare	1.0 e-148

00731	2559	697	Collagen pro-α chain	Haliotis discus	Pacific abalone	9.5 e-31

00765	1668	647	Enolase	Loligo pealei	Long-finned squid	1.5 e-184

17466	2025	547	ATP synthase sub-unit α	Pinctada fucata	Pearl oyster	1.9 e-243

02034	1166	544	Collagen type IV α6	Ciona intestinalis	Sea squirt	2.0 e-13

17241	1652	544	Troponin T	Patinopecten yessoensis	Yesso scallop	1.9 e-32

16715	1674	505	B cell translocation gene	Crassostrea gigas	Pacific oyster	2.1 e-36

17817	3029	477	Poly adenylate binding protein	Bos taurus	Cow	7.6 e-196

17035	3832	466	Phosphoenolpyruvate carboxylase	Crassostrea gigas	Pacific oyster	1.1 e-270

00554	1333	455	Ornithine decarboxylase	Haliotis diversicolor	Abalone	6.9 e-63

01359	2332	449	Tyrosinase	Sepia officinalis	Cuttlefish	9.3 e-47

01057	2938	438	Arginine kinase	Carbicula japonica	Shijimi clam	5.1 e-267

00449	1698	428	Voltage gated potassium channel complex	Mus musculus	Mouse	7.2 e-11

17467	535	421	Stress associated endoplasmic reticulum protein (SERP2)	Bos taurus	Cow	3.0 e-21

00029	2433	411	Transport protein SEC1 subunit α	Culex quinquefasciatus	Mosquito	4.4 e-235

01548	1543	405	Calponin/transgelin	Haliotis discus	Pacific abalone	1.7 e-34

00054	1486	399	Mitochondrial carrier protein, putative ADP/ATP translocase	Lepeophtheirus salmonis	Copepod sea louse	7.3 e-112

00562	3798	396	Thymosin β	Triatoma infestans	Chagas insect disease vector	4.0 e-19

00500	1985	395	Adipose differentiation-related protein	Anas platyrhynchos	Mallard duck	2.5 e-42

00119	966	388	40 s ribosomal protein S2	Urechis caupo	Echiuran worm	1.2 e-109

02431	804	382	60 s ribosomal protein L15	Ctenopharyngodon idella	Grass carp	7.8 e-76

00103	2028	374	NADH-ubiquinone oxidase	Lophiotoma cerithiformis	Conoidean gastropod	6.6 e-65

01042	1310	359	Y-box factor homolog	Aplysia californica	California sea hare	6.1 e-43

00753	3170	357	Vacuolar ATP synthase	Salmo salar	Atlantic salmon	2.2 e-49

00168	1820	356	Myosin heavy chain	Mytilus galloprovincialis	Mediteranean mussel	1.6 e-191

17000	912	354	Ribosomal protein L3	Spodoptera frugiperda	Fall armyworm	3.9 e-113

00730	1839	351	ATP synthase sub unit β	Pinctada fucata	Pearl oyster	1.5 e-111

17045	513	341	Ribosomal protein L28	Sipunculus nudus	Marine worm	2.2 e-39

16762	1674	325	Ubiquitin-conjugating enzyme	Rhipicephalus sanguineus	Brown dog tick	2.4 e-13

01081	1740	325	Calponin	Mytilus galloprovincialis	Mediteranean mussel	6.4 e-51

01704	1585	324	α macroglobulin	Macrobrachium rosenbergii	Giant river prawn	1.6 e-40

00954	1609	323	Catalase	Chlamys farreri	Japanese scallop	7.9 e-216

01079	2064	318	Troponin	Patinopecten yessoensis	Yesso scallop	3.4 e-36

01055	4390	317	Mannan-binding lectin-associated serine protease	Cyprinus cario	Common carp	4.4 e-32

05926	594	313	40 s ribosomal protein S11	Lineus viridis	Nemertean	3.3 e-61

00567	614	311	GABA (A) receptor associated protein	Brachiostoma belcheri	Amphioxus	7.7 e-57

17744	1394	309	Prosaposin	Danio rerio	Zebrafish	1.8 e-10

17082	604	307	Actin	Podocoryne carnea	Hydrozoan	3.2 e-42

00083	758	306	Fructose-biphosphate aldolase	Haliotis discus	Pacific abalone	8.0 e-89

Sequence similarity searching of the GenBank non-redundant database with BLAST produced matches against only 3,098 of the contigs using a < 1e^-10 ^cut off value. This poor level of sequence similarity matching (17%) has also been noted in previous work on this species (Truebano et al, submitted) and is due to a lack of sequences from a closely related mollusc species in the databases. This is reflected in the number of different species that show sequence matches against our data; Table 1 comprises 39 BLAST sequence similarity results with the best matches originating from 33 species ranging from hydrozoans and arthropods through to vertebrates. To date (25/01/10) there are only 25,032 nucleotide sequences, 195,275 ESTs, 14,507 proteins and 356 genes from the class Bivalvia in the public databases http://www.ncbi.nlm.nih.gov and these are dominated by entries from *Mytilus *and *Crassostrea *species. At the sub-class level, the number of nucleotide and protein entries are 86 and 19 respectively, which is further reduced to 24 and 16 at the family level. The genbank non-redundant database [53] is one of the best annotated sources for comparative *in silico *gene analyses. However, of potential use, in terms of EST verification and gene mining are other less well annotated sources of molluscan sequence data, such as the sequenced genome of the gastropod snail (*Lottia gigantea*) and 454 data from *Mytilus *species [54]. These comprise larger molluscan datasets than found in genbank, but BLAST sequence similarity searches using a <1e-10 cut off value merely emphasized the evolutionary distance between the molluscs studied. For example, just over 2% of the *Laternula *contigs matched the ESTs and EST clusters produced from *Lottia*, although this increased to 17.5% against the Lottia filtered gene set. Less than 1% of the *Laternula *contigs matched the *Mytilus *mantle-specific 454 libraries and the 42,364 ESTs from *M. californianus *in GenBank. Hence there are no species closely related to *L. elliptica *with large amounts of sequence data in the public domain and therefore our data significantly increases resources in this area and provides an important source of comparative data for other Molluscan species.

### Highly expressed sequences

**Figure 2:**
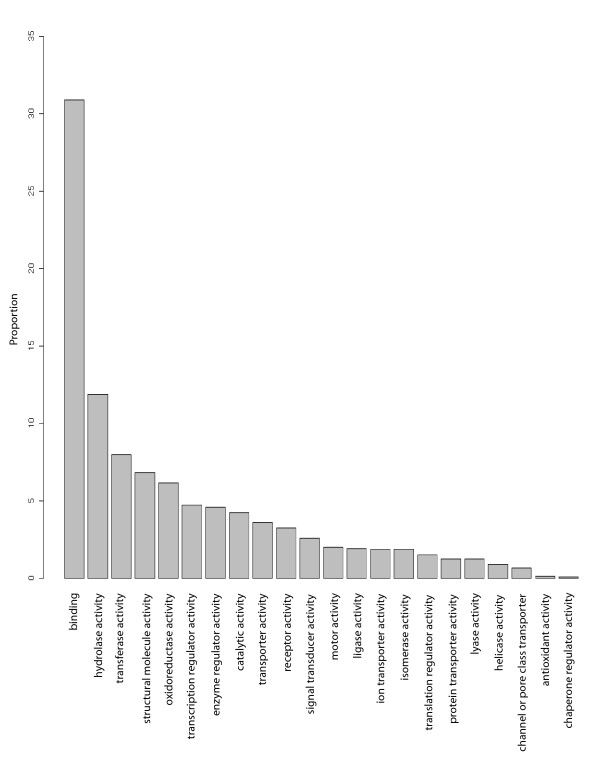
**GO categories of genes identified in *L. elliptica *data by BLAST sequence similarity searching**.

The most commonly expressed genes in the *Laternula *dataset comprise various functional classes, which is reflected in the overall GO classifications (Figure 2). As stated previously, the edge of the mantle comprises three folds and the periostracum with the tissue for this transcriptome analysis taken from a cross section across all layers. BLAST sequence similarity searches revealed a wide range of diverse functions among the most commonly expressed genes (contigs comprising over 300 individual sequences) (Table 1) reflecting the complex contractile and secretory nature of this organ.

The mantle, whilst not a muscle *per se*, is contractile and hence many of the highly expressed sequences consist of structural or muscle-related genes, such as actin, collagen, troponin, calponin, adipose differentiation-related protein and myosin [55], although some e.g. collagen, may also be involved in shell synthesis [56]. Interestingly, the most commonly expressed sequence is that of a MAP kinase interacting serine threonine protein kinase (Mnk1). This gene is a transcriptional and translational regulator of mRNA, in particular acting via the phosphorylation of the elongation initiation factor (EIF4E), which is an important modulator of cell growth and proliferation [57]. Studies in *Aplysia *have shown Mnk1 to be a negative regulator of cap-dependant translation in neurons [58], whilst in other species it has also been shown to bind stress activated p38 and may play a role in response to environmental stress [59]. The role of this gene in cell growth links with the identification of the B cell translocation gene (also involved in cell differentiation) and the Y-box factor homologue (a transcriptional and translational regulator of mRNA) [60], indicating that the mantle is an area of continual growth.

From the above, the mantle is clearly a metabolically and transcriptionally active tissue. This is further exemplified by the presence of ATP synthases, an ADP/ATP translocase, NADH-ubiquinone oxidase, genes from the glycolysis pathway, ribosomal RNAs and arginine kinase. The latter is a phosphagen kinase and these enzymes are prevalent in systems with fluctuating energy demands, acting as an energy buffering system [61] and also as an energy shuttle delivering ATP generated by mitochondria to high energy requiring processes, such as membrane turnover and potentially shell deposition [62]. Whilst the phosphagen kinases are a multigene family, arginine kinase is the only form of this gene in arthropods and molluscs [63]. It also has other functions such as buffering intracellular pH which would be important in the extrapallial space with the supersaturation of shell matrix components, including calcium ions.

**Figure 3:**
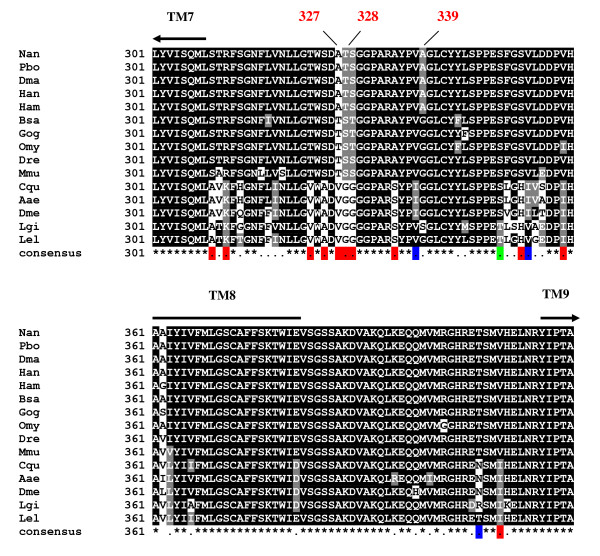
**Amino acid alignment of the region between transmembrane (TM) regions 7 and 9 of the α sub-unit of SEC61**. Putative cold-adapted amino acid substitutions are indicated at positions 327, 328 and 339. Invertebrate-specific substitutions are labelled in red on the consensus line, insect-specific substitutions labelled in blue and the single potential bivalve-specific substitution labelled in green. Species abbreviations and accession numbers: Nan: *Notothenia angustrata *(Q8AY35); Pbo: *Pagothenia borchgrevinki *(Q8AY36); Dma: *Dissostichus mawsoni *(AY113841); Han: *Harpagifer antarcticus *(Q7T278); Ham: *Hemitripterus americanus *(Q8AY34); Bsa: *Boreogadus saida *(Q8AY33); Gog: *Gadus ogac *(Q8AY32) (all cold-adapted); Omy: *Onchorhynchus mykiss *(Q98SN9); Dre: *Danio rerio *(Q90ZM2); Mmu: *Mus musculus *(P61620); Cqu: *Culex quinquefasciatus *(B0WNA0); Aae: *Aedes aegypti *(Q17CM3); Dme: *Drosophila melanogaster *(Q8STG9). Lgi: *Lottia gigantea *(cluster: >jgi|Lotgi1|194715|estExt_Genewise1.C_sca_610223 from sequences: 4236761:1772, 4236761:2059 and 4236761:4476 extracted from http://genome.jgi-psf.org/Lotgi1/Lotgi1.home.html; Lel: *Laternula elliptica*, contig00029.

The secretory nature of the mantle tissue requires a number of membrane transport proteins, represented in our limited identifications by a component of a voltage gated potassium channel complex, a V-type ATP synthase, which may transport solutes and lower pH in organelles, prosaposin and the endoplasmic reticulum transport protein SEC61 α sub-unit. The latter protein has been functionally studied in yeast and shown to play a crucial role in translocation of secretory polypeptides across the endoplasmic reticulum membrane [64]. Protein alignments of SEC61 α sub-unit from cold (Polar) and temperate fish species identified a number of putative cold adaptive amino acid substitutions [64]. The *L. elliptica *data contained the full length sequence of this gene and alignment with other species, specifically the fish forms [64] showed that *L. elliptica *does not have the proposed teleost cold water-specific amino acid modifications at positions 327, 328 and 339 in the loop between transmembrane regions 7 and 8 (Figure 3). One hypothesis, at the time, was that these changes were not adaptive, but inherited from a common fish ancestor and our data would appear to substantiate the latter hypothesis. Indeed over the stretch of 120 amino acids shown (Figure 3), the addition of *L. ellipitica*, the temperate bivalve *L. gigantea *and three insects to the fish alignment [64] indicates that within this stretch alone, there are 11 putative invertebrate-specific substitutions, 3 substitutions specific to the insects and one restricted to the 2 mollusc species. The implications of these changes cannot be quantified without functional studies, however, our Antarctic invertebrate dataset provides a significant resource for further investigation of gene and protein evolution in cold adapted metazoan species.

Animals living in constant cold temperatures could initially be thought to be more vulnerable to damage by reactive oxygen species, due to slow cell and protein turnover rates and the consequent accumulation of oxidised proteins [12,65]. However, *L. elliptica *is being used as a model for ageing studies as this organism appears to have uncoupled the ageing process and antioxidant production. It has a higher antioxidant capacity compared to shorter lived temperate clams, with a constant requirement for stable antioxidant status until old age [11,12]. Enzyme assays show that catalase, in particular, remains at a constant level throughout its lifetime and this is exemplified by the expression of catalase in the genome of adults of this species. The stress associated endoplasmic reticulum protein (SERP2) is also highly expressed and this may be linked to antioxidant capacity or it may protect unfolded target proteins against degradation and facilitate correct glycosylation. As a member of the RAMP4 family (ribosome associated membrane proteins) it may also be involved in the stabilisation of membranes in response to stress. It is known that there are problems folding proteins at low temperatures [66] and to date, of the few Antarctic marine species investigated, the majority do not exhibit the classical heat shock protein (HSP) stress response. Indeed, several species express the inducible form of HSP70 permanently, possibly as a measure towards more efficient folding of proteins at low temperatures [21] and the expression of SERP2 may contribute towards this "extra" required function and form part of a "preparative defence" strategy against the cold [67].

The final category of highly expressed genes comprises those involved in immune function, e.g. thymosin β [68], α macroglobulin and a mannan-binding lectin associated serine protease, which is a complement control module. The reason for this up-regulation may be slightly more complex than it initially appears. The mantle edge is in constant contact with the external environment and hence there will be permanent challenges to the immune system. This is compounded in the Rothera population of *L. elliptica *by a significant amount of physical damage (Harper, pers comm). These almost certainly will require the enhanced expression of immune-related genes, as the external protection of the shell is compromised, along with matrix deposition for shell repair. However, physical damage is a stressor in its own right which along with the effect of the cold environment (another potential stressor) may induce changes in the immune system as stress and the immune system have been found in many species to be inextricably linked [69].

### Putative shell deposition transcripts

The formation of the skeleton in animals is well conserved and frequently involves calcification of a macromolecular network of proteins, lipids and polysaccharides. In molluscs the mantle is the source of matrix proteins and other secreted factors which promote the extracellular assembly of the shell. Relatively few matrix proteins contributing to the shell in molluscs have been identified and most of the studies so far have focused on single proteins such as Asprich, lustrin A, perlustrin and calconectin, whilst other proteins involved in calcium deposition include carbonic anhydrase [36,38-40,70-72]. In a recent study, 331 randomly selected clones from a cDNA library of the juvenile mantle of tropical abalone (*Haliotis asinina*, Linnaeus) were sequenced [73]. The authors reported that 26% of the genes encoded secreted proteins and of the 106 unigenes identified 15 were involved in trafficking and mineral binding, mechanisms which they suggested probably contribute to construction of the shell. In the present study a conservative estimate using the GO cellular component annotation of known genes suggests 40% of the transcripts are likely to be secreted proteins. A comparison of the transcriptome of the mantle from adult *L. elliptica *with the cDNA isolated from juvenile tropical abalone mantle [73] revealed relatively poor conservation, with only 31 of the *Haliotis *sequences sharing significant sequence similarity with the *Laternula *transcripts. This may be due to either the disparity in sample sizes or maturity stage of the animals, rather than evolutionary distance, as BLAST sequence similarity searching of all 6778 *Haliotis asinina *sequences in GenBank produced a higher match with 728 *Laternula *contigs matching 1435 *Haliotis *sequences (21%). Indeed there were relatively few matches to ESTs from libraries generated specifically to study nacre building gene sets in *Haliotis asinina *and the bivalve *Pinctada maxima *(6,122 and 6,737 ESTs respectively) indicating the divergence in biomineralisation processes between these two different molluscs [56]. This was further highlighted in the *Haliotis/Pinctada *study, where there was very little overlap between even the most highly expressed genes and addition of the results from the *Laternula *and *M. galloprovincialis *datasets substantiate this (Table 2). Hence there is a requirement to understand shell deposition in a variety of molluscs and not just work on a single model species, particularly where there is a requirement to understand environmental effects.

**Table 2:** The ten most commonly expressed sequences (in order of abundance) in mantle tissue from 4 bivalves.

*Laternula elliptica*	*Haliotis asinina*	*Pinctada maxima*	*Mytilus galloprovincialis*
map kinase interacting serine threonine protein kinase.	-	shematrin	-

collagen pro-α chain	Elongation factor-1α	shematrin	-

enolase	cytochrome c oxidase 1	16 s ribosomal protein	Phospholipase

-	-	KRMP-8, glycine rich structural protein	-

-	ferritin	Elongation factor-1α	NADH dehydrogenase subunit 4

-	collagen	actin	-

ATP synthase sub-unit α	cytochrome c oxidase 1	KRMP-8, glycine rich structural protein	-

collagen type IV α6	-	N14 matrix protein	NADH dehydrogenase subunit 4

troponin T	-	-	NADH dehydrogenase subunit 4

-	-	paramyosin	NADH dehydrogenase subunit 4

The *Laternula *and *Mytilus *sequences arise from 454 data [*Mytilus *data: J. Gilbert, pers comm.], the *Haliotis *and *Pinctada *data are from EST library sequencing [56].

Several of the most highly expressed genes in our dataset are almost certainly involved in shell deposition, including tyrosinase. The periostracum is secreted as a soluble precursor (the periostracin) and this is then cross-linked by o-diphenols and tyrosinase (or phenoloxidases) to form an insoluble periostracum [74,75]. Tyrosinase can also be involved in pigment formation in the prismatic layer and evidence from the pearl oyster demonstrates several different paralogues of tyrosinase which are involved in these different functions [76,77]. However, in order to discover genes within our dataset that are likely to play a role in shell deposition and calcium regulation, we searched the literature to generate an in-house database of proteins involved in extracellular matrix (ECM) formation and calcium homeostasis in metazoans (Supplemental Tables 1 and 2). Numerous transcripts were identified; hence the following section will give only a brief outline of the putative role of the more abundant transcripts.

The presence of putative transcripts for carbonic anhydrase in *L. elliptica *mantle is unsurprising as this protein was first identified in the shell in 1948 [78] and it has subsequently been implicated in matrix mineralisation by generating an acidic environment through the conversion of respiratory CO_2 _into HCO_3 _in the presence of water [38]. Putative transcripts for the matricellular glycoprotein, secreted protein acidic rich in cystein (SPARC, a basal membrane component) were also identified. This trimodular protein promotes proper assembly and maturation of the matrix scaffold and is highly conserved in animal phyla [79]. In vertebrates the latter is achieved in part through the interaction of SPARC with fibril forming collagens (I, II, III and V) [80,81] and although it is necessary to conduct further work to better characterize these transcripts, orthologues of collagen I, II and V were identified.

Additional transcripts identified in the *L. elliptica *mantle transcriptome potentially implicated in ECM formation/turnover in metazoans include the thrombospondins, which are a family of large, secreted, multi-modular, calcium-binding glycoproteins which appear to interact with collagens and integrins and have been implicated in skeletal disorders in mammals [82]. Transcripts for tenascin, a large glycoprotein containing several fibronectin III type repeats and implicated in cell adhesion in chordates was identified in *L. Elliptica *[83]. Interestingly, several transcripts for metalloproteinase 1 (collagenase 1) which are important in extracellular matrix turnover were also identified. It is apparent even from this brief consideration that numerous homologues of genes identified in the ECM of the vertebrate skeleton are also present in the mantle transcriptome. Future work will permit a more precise characterization of the localization and function of these mantle transcripts and hence provide a better understanding of shell formation. This will be essential for ecophysiological studies.

### The clam orphan calcium regulatory receptors

The *L. elliptica *contig11573 (283 bp) and contig14182 (252 bp) nucleotide reads share the highest sequence similarity for the N-terminal region and TM2 to TM3 of the metazoan parathyroid hormone receptor (PTHR) and calcitonin/calcitonin Gene-related peptide (CLR/CGRPR) receptors, repectively (Table 3). In vertebrates these receptors are important mediators of the action of the calcitropic factors, calcitonin (CT) and parathyroid hormone (PTH) which stimulate respectively, calcium uptake and bone formation and calcium release for serum and bone turnover. In invertebrates, putative protostome CLR/CGRPR transcripts that remain to be functionally characterised have previously been identified [47,84]. Several scaffolds were identified in the *Lottia *genome assembly which shared high sequence similarity (e^-18 ^- e^-13^) to the *L. elliptica *contigs similar to PTHR/CLR. In molluscs, a CLR/CGRP, expressed in the mantle of the eastern oyster *Crassostrea virginica *(JC8022) [85] has been isolated and found to be functionally conserved with the vertebrate orthologues. Despite the recent identification of a prototype of vertebrate CT/CGRP peptide ligand in the ascidian *Ciona intestinalis *[86] no transcripts which are orthologues of CT or PTH have been identified in *Laternula *and the functional significance of the Antarctic bivalve receptors remains to be established.

**Table 3:** Best database matches of the *Laternula *sequences to the family 2 GPCRs.

Read	Length (bp)	Sequence similarity
contig11573	283	PREDICTED: similar to parathyroid hormone receptor [Nasonia vitripennis] 4e-17 calcitonin receptor [Culex quinquefasciatus] 2e-14

contig14182	252	PREDICTED: similar to calcitonin receptor, partial [Acyrthosiphon pisum] 9e-10

**Figure 4:**
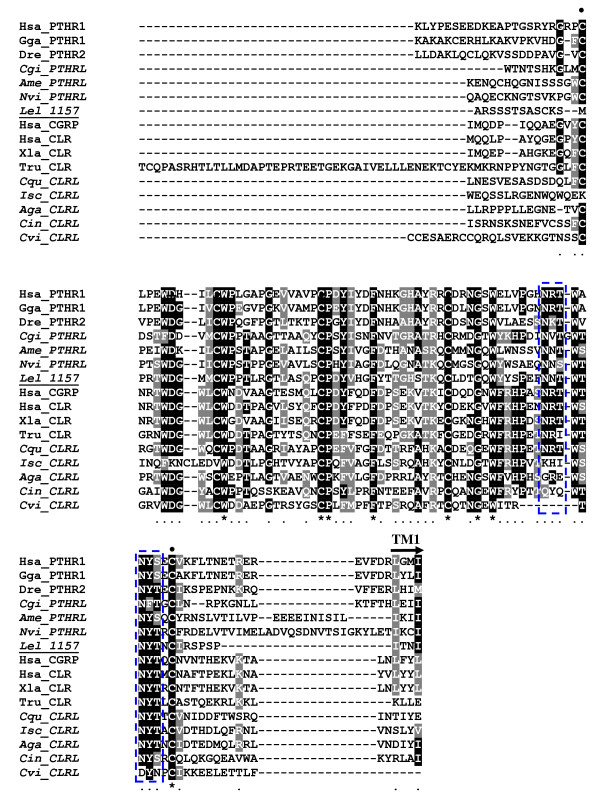
**Multiple sequence alignment of the putative PTH/CALR receptor in *L. elliptica *contig 11573 (Lel_11573) with the N-terminal region of the putative metazoan homologues**. The sequence alignment starts from the beginning of the *Laternula *fragment. Conserved cysteine residues are indicated by dots "•" and the Aspartic acid (D) residue within the N-terminal sequence motif C-x(4)-D-x(3,4)-C-Wx(11,12)-C-P involved in CLR/RAMP/ligand interactions indicates by a cross "+". The beginning of receptor TM1 region is indicated by an arrow and the localisation of putative glycosylation sites (NXT/S) indicated by blue dashed boxes. Amino acid conservation in the alignment is colour coded and black shaded columns mean total residue conservation. Accession numbers of the sequences used in the alignment are: Human (Hsa, PTHR1 NP_000307; CALR NP_001158209; CGRP NP_005786); Chicken (Gga, XP_418507); Zebrafish (Dre, AAI62580); *Xenopus laevis *(Xla, NP_001080206); *Takifugu rubripes *(Tru, NP_001098689); *Crassostrea virginica *(Cvi, JC8022 (est)); *Ciona intestinalis *(Cin, BAI63096); *Crassostrea gigas *(Cgi, AM858508); *Culex quinquefasciatus *(Cqu, XP_001864896); *Anopheles gambiae *str. PEST (Aga, XP_321982); *Ixodes scapularis *(Isc, XP_002414039); *Apis mellifera *(Ame, XP_001122670); *Nasonia vitripennis *(Nvi, XP_001605780). The predicted invertebrate proteins are marked in italics and were included for comparison with the bivalve (*L. Elliptica; *Lel) deduced amino acid sequence of contig 11573 which is highlighted in bold.

**Figure 5:**
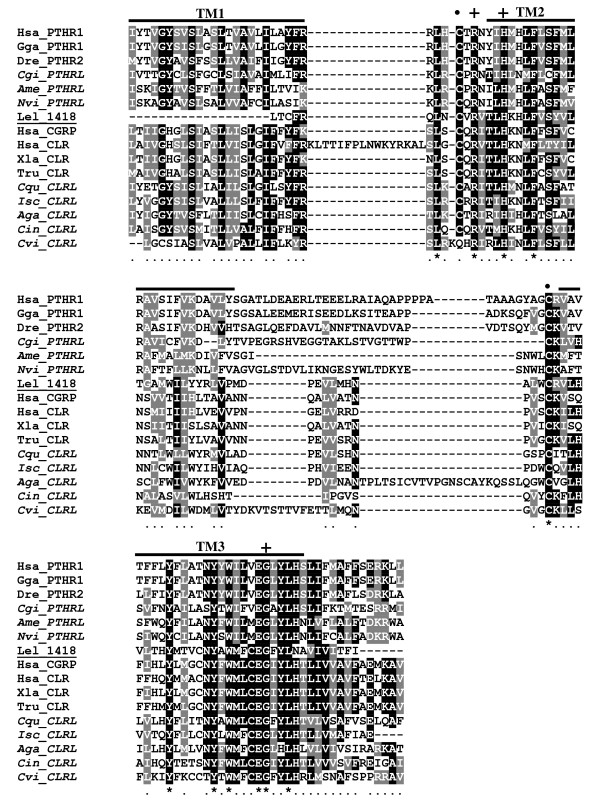
**Multiple sequence alignment of the putative PTH/CALR receptor in *L. elliptica *contig 14182 (Lel_14182) with the TM domain region of putative metazoan homologues**. The localization of TM1, TM2 and TM3 are indicated by lines and ICL 1 and 2 (intracellular loop) and ECL1 (extracellular loop) are named. Conserved cysteines are indicated by dots "•" and amino acid residues involved in G*s *coupling are marked with a cross "+". Amino acid conservation in the alignment is colour coded and black shaded columns mean total residue conservation. Accession numbers of sequences used in the alignment are the same as for Figure 3 and predicted invertebrate proteins are marked in italics and the deduced amino acid sequence from *L. elliptica *(Lel) is highlighted in bold.

Despite the relatively short sequence of the family 2 B1 G-protein coupled receptor transcripts in *L. elliptica *it was possible to identify conserved amino acid motifs implicated in receptor conformation and ligand affinity in metazoan orthologues (Figures 4 and 5) [84]. The characteristic N-terminal motif for interacting with receptor activity-modifying proteins (RAMPs) was identified in contig11573 [87]. RAMPs are known to modulate GPCRs and in vertebrates they define the specificity of the CLR by modifying its affinity for the ligand. Putative RAMP transcripts were not identified in the present study and it remains to be established if the bivalve family 2 B1 GPCRs are modulated in a similar way to those in vertebrates.

## Conclusions

Comprehensive 454 pyrosequencing of mantle tissue from the Antarctic clam **(***Laternula elliptica*) has produced a transcriptome of 18,290 contigs. In spite of a low level of putative gene identifications (17%, produced via database sequence similarity searching), it was possible to identify a considerable number of transcripts putatively related to shell deposition (e.g. tyrosinase and SPARC). This was via similarity to annotated sequences from other molluscs in the databases or to genes known to be involved in skeletal formation in vertebrates. In particular, 2 putative members of family 2 GPCRs were identified which share the highest sequence similarity to the metazoan parathyroid hormone (PTH) and calcitonin/calcitonin Gene-related peptide (CLR/CGRPR) receptors. In vertebrates these genes are important mediators of the action of the calcitropic factors, calcitonin (CT) and parathyroid hormone (PTH). This dataset, whilst being the first 454 sequence to be generated from an Antarctic invertebrate, also provides a significant resource for comparative studies into protein cold adaptation and the processes of shell deposition, in particular the reaction of the shell secretome to environmental stress in the form of climate change effects. Both avenues will be explored in the future within our laboratory, both in *Laternula *and economically important model temperate mollusc species, such as *Crassostrea gigas *and *Mytilus galloprovincialis*.

## Methods

### Animal sampling

All animals used in experimental work were collected at Rothera Research Station, Adelaide Island, Antarctic Peninsula (67° 4' 07" S, 68° 07' 30" W) by SCUBA divers during the austral summer at depths of 10-15 m. The animals were immediately returned to the laboratory where they were maintained in a through-flow aquarium with a temperature of 0.6 ± 0.3°C, under a simulated natural light:dark cycle. All animals were mature adults, with a range of shell sizes between 50.1-83.5 mm. As shell length is related to animal age: surface aging estimates using growth rings produced an mean age of 8.3 years (SE mean 0.207) with a range from 6-14 years and a median of 8 years (S. Morley pers comm). Mantle tissue was dissected from the animals and cross sections comprising all 3 folds and the periostracum were immediately flash frozen in liquid nitrogen for later RNA extraction.

### RNA isolation and cDNA production

Mantle RNA was extracted from 24 animals using a modified TRI reagent protocol. After homogenization in Tri Reagent (Sigma) and chloroform extraction, the samples were subjected to a lithium chloride precipitation step. RNA was precipitated using a 1:1 isopropanol:saline solution (0.8 M sodium citrate and 1.2 M NaCl) and after resuspension, the RNA was subjected to a further precipitation using 250 μl 7.5 M LiCl. The extractions were further cleaned using RNeasy mini kit columns (Qiagen, Crawley, Sussex, UK) following manufacturer instructions in order to eliminate LiCl and salt residues. 5 μg of RNA was PCR amplified using the protocol described in [88] prior to preparation for the 454 run. Samples were nebulised at 30psi for one minute and subsequently purified with Ampure (Agencourt) to produce fragments 300 bp and above. The ends were polished and the 454 titananium adapters containing specific MID sequences were attached. Fragments containing both a and b adapters were selected and quantified. Libraries were amplified by emulsion PCR, beads recovered and enriched and placed on a picotiter plate for sequencing by the 454 procedure.

### 454 Assembly and Analysis

The raw data comprised 1,034,155 reads. Crossmatch (P. Green, unpublished) was then applied to screen for adaptor sequences and other artifacts of the pyrosequencing procedure and also vector sequences using the UniVec database http://www.ncbi.nlm.nih.gov/VecScreen/UniVec.html. Stripping the masked sequence from the ends and removing reads with masked sequence in the middle resulted in 778,629 sequences that were entered into the Newbler program [51] for assembly. This resulted in 18,290 contigs. All singletons were discarded. Files containing the reads have been submitted to the National Center for Biotechnology Information Short Read Archive (accession number SRA011054). The mapping facility of Newbler was applied to the assembly to determine the number of SNPs, and Phobos [89] was used for microsatellite discovery. The contigs were then searched for sequence similarity using BLAST [90] against the genbank non-redundant database [53] and unannotated data from other bivalve species: the gastropod snail: *Lottia gigantea *http://genome.jgi-psf.org/Lotgi1/Lotgi1.home.html and the *Mytilus *454 mantle-specific datasets (4442949.3: *M. galloprovincialis *mantle unassembled and 4442954.3 *M. edulis *mantle unassembled) [54] lodged under the MG-RAST database: Meta Genome Rapid Annotation using Sub-system Technology http://metagenomics.nmpdr.org/) [91]. The Gene Ontology (GO) [92] mappings were determined by an in-house database on all Swissprot and Trembl [93] BLAST scores below a threshold of 1e-10. Sequence manipulation was carried out using the EMBOSS suite of programmes [94]. Sequences were clustered using ClustalW [95] and the alignments displayed using BoxShade v3.21 [96].

## Authors' contributions

MSC progressed the original project idea through to 454 analysis, organised the sequencing, provided the manual assembly verification, analysis of the most commonly expressed transcripts and wrote this section of the manuscript, along with developing the initial concept of the paper and co-ordinating input for the final manuscript. MAST performed all the 454 computational analysis, including installation, development of the 454 pipeline and collation/liaison of datasets. JCRC, FAV and DMP developed and produced the in-house databases on genes putatively involved in calcium regulation and shell deposition and the family 2 GPCRs. JCRC specifically analysed the family 2 GPCR data and FAV analysed the calcium regulation and shell deposition transcripts. DMP supervised this part of the analysis and wrote the section of the manuscript specifically relating to this, providing additional input into final editing of the manuscript. LSP assisted with development of the initial project concept, provided laternula material, physiological input and was involved in data interpretation, manuscript preparation and editing. All authors have read and approved the final manuscript.

## Supplementary Material

Additional file 1 **Table S1**. List of genes and accession numbers comprising in-house database of proteins involved in extracellular matrix (ECM) formation and calcium homeostasis in metazoans. Click here for file

Additional file 2 **Table S2**. List of genes and accession numbers comprising in-house database of the family 2 GPCRs. Click here for file

Additional file 3 **Table S3**. Microsatellite repeats found in excess of 7 copies per repeat unit in *L. elliptica *data. Click here for file

Additional file 4 **Table S4**. Variant nucleotides (SNPs/INDELS) found in *L. elliptica *data. Click here for file
