# Supplementary material for: Insights into shell deposition in the Antarctic bivalve Laternula elliptica: gene discovery in the mantle transcriptome using 454 pyrosequencing
Source: BMC Genomics. 2010 Jun 8;11:362. doi: 10.1186/1471-2164-11-362 (PMC2896379; doi:10.1186/1471-2164-11-362)
Supplement: Additional file 1 — Table S1. List of genes and accession numbers comprising in-house database of proteins involved in extracellular matrix (ECM) formation and calcium homeostasis in metazoans. [file 1471-2164-11-362-S1.PDF]

Code	Gene ID	Specie	Accession #
FARO_FV1	Thrombospondin_1 (THSB1)	Homo sapiens	NM_003246.2
FARO_FV2	Thrombospondin_2 (THSB2)	Homo sapiens	NM_003247.2
FARO_FV3	Thrombospondin_3(THSB3)	Homo sapiens	NM_007112.3
FARO_FV4	Thrombospondin_4(THSB4)	Homo sapiens	NM_003248.3
FARO_FV5	COMP(THBS5)	Homo sapiens	NM_000095.2
FARO_FV6	laminin1(LAMA1)	Homo sapiens	NM_005559.2
FARO_FV7	laminin2(LAMA2)	Homo sapiens	NM_000426.2
FARO_FV8	laminin3(LAMA3)	Homo sapiens	NM_000227.2
FARO_FV9	laminin4(LAMA4)	Homo sapiens	NM_002290.2
FARO_FV10	laminin5(LAMA5)	Homo sapiens	NM_005560.3
FARO_FV11	laminin_beta1(LAMB1)	Homo sapiens	NM_002291.1
FARO_FV12	laminin_beta2(LAMB2)	Homo sapiens	NM_002292.2
FARO_FV13	laminin_beta3(LAMB3)	Homo sapiens	NM_000228.2
FARO_FV14	laminin_beta4(LAMB4)	Homo sapiens	NM_007356.1
FARO_FV15	laminin_gamma1(LAMC1)	Homo sapiens	NM_002293.2
FARO_FV16	SPARC	Homo sapiens	NM_003118.2
FARO_FV17	COL1A1	Homo sapiens	NM_000088.2
FARO_FV18	COL4A3	Homo sapiens	NM_000091.2
FARO_FV19	COL4A6	Homo sapiens	NM_001847.1
FARO_FV20	COL5A1	Homo sapiens	NM_000093.2
FARO_FV21	COL5A3	Homo sapiens	NM_015719.2
FARO_FV22	COL6A1	Homo sapiens	NM_001848.1
FARO_FV23	COL6A2	Homo sapiens	NM_001849.2
FARO_FV24	COL6A3	Homo sapiens	NM_004369.2
FARO_FV25	COL7A1	Homo sapiens	NM_000094.2
FARO_FV26	COL8A1	Homo sapiens	NM_001850.3
FARO_FV27	COL8A2	Homo sapiens	NM_005202.1
FARO_FV28	COL9A1	Homo sapiens	NM_001851.3
FARO_FV29	COL11A1	Homo sapiens	NM_080629.1
FARO_FV30	COL11A2	Homo sapiens	NM_080679.1
FARO_FV31	COL12A1	Homo sapiens	NM_004370.4
FARO_FV32	COL14A1	Homo sapiens	NM_021110.1
FARO_FV33	COL15A1	Homo sapiens	NM_001855.2
FARO_FV34	COL16A1	Homo sapiens	NM_001856.2
FARO_FV35	COL18A1	Homo sapiens	NM_030582.2
FARO_FV36	COL19A1	Homo sapiens	NM_001858.4
FARO_FV37	COL24A1	Homo sapiens	NM_152890.4
FARO_FV38	COL27A1	Homo sapiens	NM_032888.2
FARO_FV39	FN1	Homo sapiens	NM_002026.2
FARO_FV40	KAL1	Homo sapiens	NM_000216.1
FARO_FV41	ADAMTS1	Homo sapiens	NM_006988.3
FARO_FV42	MMP1	Homo sapiens	NM_002421
FARO_FV43	MMP10	Homo sapiens	NM_002425.1
FARO_FV44	MMP11	Homo sapiens	NM_005940.3
FARO_FV45	MMP12	Homo sapiens	NM_002426.2

FARO_FV46	MMP13	Homo sapiens	NM_002427.2
FARO_FV47	MMP14	Homo sapiens	NM_004995.2
FARO_FV48	MMP15	Homo sapiens	NM_002428.2
FARO_FV49	MMP16	Homo sapiens	NM_005941.3
FARO_FV50	MMP2	Homo sapiens	NM_004530.2
FARO_FV51	MMP3	Homo sapiens	NM_002422.3
FARO_FV52	MMP7	Homo sapiens	NM_002423.3
FARO_FV53	MMP8	Homo sapiens	NM_002424.1
FARO_FV54	MMP9	Homo sapiens	NM_004994.2
FARO_FV55	MMP17	Homo sapiens	NM_016155.3
FARO_FV56	MMP20	Homo sapiens	NM_004771.3
FARO_FV57	MMP24	Homo sapiens	NM_006690.3
FARO_FV58	MMP26	Homo sapiens	NM_021801.3
FARO_FV59	paraplegin(SPG7)	Homo sapiens	NM_003119.2
FARO_FV60	TIMP1	Homo sapiens	NM_003254.2
FARO_FV61	TIMP2	Homo sapiens	NM_003255.4
FARO_FV62	TIMP3	Homo sapiens	NM_000362.4
FARO_FV63	versican(CSPG2)	Homo sapiens	NM_004385.2
FARO_FV64	CTGF	Homo sapiens	NM_001901.1
FARO_FV65	ECM1	Homo sapiens	NM_004425.2
FARO_FV66	HAS1	Homo sapiens	NM_001523.1
FARO_FV67	SPP1	Homo sapiens	NM_000582.2
FARO_FV68	TGFBI	Homo sapiens	NM_000358.1
FARO_FV69	CLEC3B_	Homo sapiens	NM_003278.1
FARO_FV70	tenascinC(TNC)	Homo sapiens	NM_002160.1
FARO_FV71	tenascinR	Homo sapiens	X98085.1_
FARO_FV72	tenascinXB(TNXB)	Homo sapiens	NM_019105.5
FARO_FV73	tenascinW	Daniorerio	AJ001423.1_
FARO_FV74	tenascinN(TNN)	Homo sapiens	NM_022093.1
FARO_FV75	vitronectin(VTN)	Homo sapiens	NM_000638.2
FARO_FV76	caveolin1(CAV1)	Homo sapiens	NM_001753.3
FARO_FV77	fibrinogenbetachain(FGB)	Homo sapiens	NM_005141.2
FARO_FV78	COL2A1	Homo sapiens	NM_001844.3
FARO_FV79	COL10A1	Homo sapiens	NM_000493.2
FARO_FV80	COL9A2	Homo sapiens	NM_001852.3
FARO_FV81	COL11A2transcriptvariant2	Homo sapiens	NM_080681.1
FARO_FV82	COL11A2transcriptvariant1	Homo sapiens	NM_080680.1
FARO_FV83	COL9A1transcriptvariant2	Homo sapiens	NM_078485.2
FARO_FV84	aggrecan1(AGC1)transcriptvariant2	Homo sapiens	NM_013227.1
FARO_FV85	aggrecan1(AGC1)transcriptvariant1	Homo sapiens	NM_001135.1
FARO_FV86	cartilagelinkprotein	Homo sapiens	X17405.1
FARO_FV87	MATN1	Homo sapiens	NM_002379.2
FARO_FV88	MATN2	Homo sapiens	NM_002380.3
FARO_FV89	MATN3	Homo sapiens	NM_002381.3
FARO_FV90	Indian_hedgehog	Homo sapiens	NM_002181.1
FARO_FV91	osteopontin (SPP1)	Homo sapiens	NM_000582.2

FARO_FV92	osteocalcin	Homo sapiens	NM_199173.2
FARO_FV93	IBSP-integrin-binding sialoprotein	Homo sapiens	NM_004967.2
FARO_FV94	ALP-1	Homo sapiens	J04948.1_
FARO_FV95	BMP2	Homo sapiens	NM_001200.1
FARO_FV96	SOX9	Homo sapiens	NM_000346.2
FARO_FV97	SOX5transcriptvariant3	Homo sapiens	NM_178010.1
FARO_FV98	SOX6transcriptvariant1	Homo sapiens	NM_017508.1
FARO_FV99	RUNX2transcriptvariant3	Homo sapiens	NM_004348.3
FARO_FV100	Sp7	Homo sapiens	NM_152860.1
FARO_FV101	SP3	Homo sapiens	NM_003111.3
FARO_FV102	FGF2	Homo sapiens	NM_002006.3
FARO_FV103	SP1	Homo sapiens	NM_138473.2
FARO_FV104	STAT1-alpha	Homo sapiens	NM_007315.2
FARO_FV105	NFATC2transcriptvariant2	Homo sapiens	NM_173091.2
FARO_FV106	NMP238	Homo sapiens	AJ010058.1_
FARO_FV107	TGFA	Homo sapiens	NM_003236.1
FARO_FV108	TGFB1	Homo sapiens	X02812.1_
FARO_FV109	TGFB2	Homo sapiens	NM_003238.1
FARO_FV110	LTBP1	Homo sapiens	NM_000627.2
FARO_FV111	LTBP1	Homo sapiens	NM_206943.1
FARO_FV112	FGF1	Homo sapiens	NM_000800.2
FARO_FV113	IGFBP1	Homo sapiens	NM_000596.2
FARO_FV114	IGF1	Homo sapiens	NM_000618.2
FARO_FV115	IGF2	Homo sapiens	NM_000612.2
FARO_FV116	Cbfa1/Runx2	Takifugu rubripes	NM_001032643.1
FARO_FV117	PDGFA	Homo sapiens	NM_002607.4
FARO_FV118	VEGF a	Homo sapiens	NM_003376.4
FARO_FV119	ENAM	Homo sapiens	NM_031889.1
FARO_FV120	BMP1	Homo sapiens	NM_001199.1
FARO_FV121	BMP3	Homo sapiens	NM_001201.1
FARO_FV122	BMP4	Homo sapiens	NM_001202.2
FARO_FV123	BMP5	Homo sapiens	NM_021073.2
FARO_FV124	BMP6	Homo sapiens	NM_001718.2
FARO_FV125	BMP7	Homo sapiens	NM_001719.1
FARO_FV126	EGF	Homo sapiens	NM_001963.2
FARO_FV127	PTH	Homo sapiens	NM_000315.2
FARO_FV128	PTHLH	Homo sapiens	NM_002820.2
FARO_FV129	BGN	Homo sapiens	NM_001711.3
FARO_FV130	PAX1	Homo sapiens	NM_006192.2
FARO_FV131	DMP1	Homo sapiens	NM_004407.1
FARO_FV132	DSPP	Homo sapiens	NM_014208.2
FARO_FV133	MEPE	Homo sapiens	NM_020203.1
FARO_FV134	S100A13	Homo sapiens	NM_005979.2
FARO_FV135	STC1	Homo sapiens	NM_003155.2
FARO_FV136	STC2	Homo sapiens	NM_003714.2
FARO_FV137	SPARCL1	Takifugu rubripes	NM_001032552.1

FARO_FV138	ACVR1	Homo sapiens	NM_001105.2
FARO_FV139	VEGF	Homo sapiens	AF022375
FARO_FV140	FGF1A	Homo sapiens	M34641
FARO_FV141	ATF2	Homo sapiens	NM_001880.2
FARO_FV142	FGFR3	Homo sapiens	NM_000142.2
FARO_FV143	PTHR1	Homo sapiens	NM_000316.2
FARO_FV144	CNP	Homo sapiens	NM_033133.4
FARO_FV145	HIF1AN	Homo sapiens	NM_017902.1
FARO_FV146	ACP5(TRAP)	Homo sapiens	NM_001611.2
FARO_FV147	TRAF(TANK)	Homo sapiens	NM_004180.2
FARO_FV148	PU1	Sus scrofa	NM_001001865.1
FARO_FV149	TRAF6	Homo sapiens	NM_004620.2
FARO_FV150	IFNB1	Homo sapiens	NM_002176.2
FARO_FV151	IFNG	Homo sapiens	NM_000619.2
FARO_FV152	AQP1	Homo sapiens	NM_198098.1
FARO_FV153	AQP2	Homo sapiens	NM_000486.3
FARO_FV154	AQP3	Homo sapiens	BC013566.1
FARO_FV155	AQP4	Homo sapiens	NM_001650.4
FARO_FV156	AQP5	Homo sapiens	BC032946.1
FARO_FV157	AQP8	Homo sapiens	NM_001169.2
FARO_FV158	AQP9	Homo sapiens	BC026258.1
FARO_FV159	AQP10	Homo sapiens	AB066105.1
FARO_FV160	AQP6	Rattus norvegicus	NM_022181.1
FARO_FV161	AQP7	Mus musculus	AB010100.1
FARO_FV162	AQP3	Xenopus laevis	AJ131847.1
FARO_FV163	AQP1	Bufo marinus	AF020620.1
FARO_FV164	AQP-t2	Bufo marinus	AF020621.1
FARO_FV165	AQP-t4	Bufo marinus	AF020623.1
FARO_FV166	aqp3	Anguilla anguilla	AJ319533.1
FARO_FV167	aquaporin	Sparus aurata	AY363261.1
FARO_FV168	AQP3	Danio rerio	BC044188.1
FARO_FV169	STC-2	Homo sapiens	AF055460.1
FARO_FV170	STC1	Danio rerio	NM_200539.1
FARO_FV171	STC2	Danio rerio	AY688947.1
FARO_FV172	MMP2	Danio rerio	NM_198067.1
FARO_FV173	MMP2	Takifugu rubripes	NM_001037869.1
FARO_FV174	MMP9	Danio rerio	NM_213123.1
FARO_FV175	VTN	Danio rerio	BC055570.1
FARO_FV176	COL2A1	Danio rerio	NM_131292.1
FARO_FV177	RUNX2b	Danio rerio	NM_212862.1
FARO_FV178	SOX9b	Danio rerio	NM_131644.1
FARO_FV179	ECaC	Danio rerio	AY325807.1
FARO_FV180	ECaC	Oncorhynchus mykiss	AY256348.1
FARO_FV181	TRPV6	Takifugu rubripes	AY232821.1
FARO_FV182	sodium/calcium exchanger	Danio rerio	NM_001037102.1
FARO_FV183	sodium/calcium exchanger	FundulusHeteroclitus	CV822288.1

FARO_FV184	sodium/calcium exchanger	FundulusHeteroclitus	CN982763
FARO_FV185	sodium/calcium exchanger	FundulusHeteroclitus	CN972776
FARO_FV186	NaPi-IIb2	Danio rerio	AF297180.2
FARO_FV187	type II Na/Pi cotransport system protein	Danio rerio	AF121796.1
FARO_FV188	sodium-phosphate cotransporter	Oncorhynchus mykiss	AY500241.1
FARO_FV189	calcium ATPase (PMCA)	Tilapiamossambica	AF236669
FARO_FV190	Liver-Sarcoplasmic/endoplasmic reticulum calci	FundulusHeteroclitus	CN958842
FARO_FV191	Heart-sarcoplasmic/endoplasmic reticulum calc	FundulusHeteroclitus	CN953792
FARO_FV192	CTR	Takifugu rubripes	NM_001105219
FARO_FV193	CTR	Takifugu obscurus	AB219840
FARO_FV194	CTR	Danio rerio	XM_683664
FARO_FV195	CTR	Homo sapiens	NM_001742
FARO_FV196	CTR	Mus musculus	NM_007588
FARO_FV197	CTR	Rattus norvegicus	NM_053816
FARO_FV198	CTR	Pan troglodytes	XM_527819
FARO_FV199	CTRL	Homo sapiens	NM_005795
FARO_FV200	CTRL	Mus musculus	NM_018782
FARO_FV201	CTRL	Rattus norvegicus	NM_012717
FARO_FV202	CTRL1	Takifugu obscurus	AB219835
FARO_FV203	CTRL2	Takifugu obscurus	AB219837
FARO_FV204	CTRL3	Takifugu obscurus	AB219838
FARO_FV205	CTRL1	Danio rerio	BC129300
FARO_FV206	CTRL3	Danio rerio	XM_001340677
FARO_FV207	RAMP1	Takifugu obscurus	AB219765
FARO_FV208	RAMP2a	Takifugu obscurus	AB219766
FARO_FV209	RAMP2b	Takifugu obscurus	AB219767
FARO_FV210	RAMP3	Takifugu obscurus	AB219768
FARO_FV211	RAMP3 splice	Takifugu obscurus	AB219769
FARO_FV212	RAMP4	Takifugu obscurus	AB219770
FARO_FV213	RAMP5	Takifugu obscurus	AB219771
FARO_FV214	CT1	Danio rerio	BC076343
FARO_FV215	CT1	Oryzias latipes	AB257081.1
FARO_FV216	CT2	Oryzias latipes	AB257082.1
FARO_FV217	CGRP2	Oryzias latipes	AB257080
FARO_FV218	CGRP1	Oryzias latipes	AB257079
FARO_FV219	PTHR1	Homo sapiens	NM_000316.2
FARO_FV220	PTHR2	Homo sapiens	NM_005048.2
FARO_FV221	CALCA	Homo sapiens	NM_001741.2
FARO_FV222	CALCB	Homo sapiens	NM_000728.3
FARO_FV223	CALCR	Homo sapiens	NM_001742.2
FARO_FV224	CRTAC1	Homo sapiens	NM_018058.4
FARO_FV225	ubiquitin	Sparus aurata	cDN01P0001G04
FARO_FV226	STAT1	Sparus aurata	cDN01P0002F02
FARO_FV227	IGF1	Sparus aurata	cDN01P0002H09
FARO_FV228	Calmodulin1	Sparus aurata	cDN01P0003H07
FARO_FV229	vitronectin	Sparus aurata	cDN01P0005N15

FARO_FV230	Col4A3	Sparus aurata	cDN01P0006C19
FARO_FV231	Biglycan	Sparus aurata	cDN02P0003F06
FARO_FV232	ubiquitin	Sparus aurata	cDN02P0004H17
FARO_FV233	PDGFRbeta2	Sparus aurata	cDN02P0005H21
FARO_FV234	COL5A3/COL5A1	Sparus aurata	cDN02P0005N01
FARO_FV235	Col14/undulin	Sparus aurata	cDN02P0005P09
FARO_FV236	calmodulin 3a	Sparus aurata	cDN02P0006M22
FARO_FV237	COL12A1	Sparus aurata	cDN03P0002D08
FARO_FV238	FGFR1	Sparus aurata	cDN03P0003N07
FARO_FV239	STAT4	Sparus aurata	cDN03P0004A16
FARO_FV240	phex	Sparus aurata	cDN03P0005E12
FARO_FV241	PDGFRbeta2	Sparus aurata	cDN04P0001C16
FARO_FV242	STAT3	Sparus aurata	cDN04P0001K20
FARO_FV243	fgfr4	Sparus aurata	cDN04P0001N21
FARO_FV244	COL5A3/COL5A1	Sparus aurata	cDN04P0002C03
FARO_FV245	COL5A3/COL5A1	Sparus aurata	cDN04P0003B02
FARO_FV246	Thrombospondin 4	Sparus aurata	cDN04P0003D14
FARO_FV247	Thrombospondin 1	Sparus aurata	cDN04P0004B09
FARO_FV248	timp-2b	Sparus aurata	cDN04P0004K04
FARO_FV249	fibronectin 1	Sparus aurata	cDN04P0005J08
FARO_FV250	fibronectin 1	Sparus aurata	cDN04P0006P16
FARO_FV251	fibronectin 1b	Sparus aurata	cDN05P0001C17
FARO_FV252	calmodulin 1b	Sparus aurata	cDN05P0005P10
FARO_FV253	FGFR1 oncogene partner 2	Sparus aurata	cDN05P0006D17
FARO_FV254	COL1A1	Sparus aurata	cDN06P0001H11
FARO_FV255	glycine-rich protein-like	Sparus aurata	cDN06P0003N19
FARO_FV256	c-Fos	Sparus aurata	cDN06P0004B14
FARO_FV257	PDGFRbeta2	Sparus aurata	cDN06P0004K11
FARO_FV258	matrix Gla protein (MGP)	Sparus aurata	cDN06P0005J19
FARO_FV259	COL1A3	Sparus aurata	cDN06P0005L21
FARO_FV260	COL2A1/COL5A2	Sparus aurata	cDN06P0006A14
FARO_FV261	cAMP-dependent transcription factor ATF-1	Sparus aurata	cDN06P0006I22
FARO_FV262	activin A receptor type II-like 1	Sparus aurata	cDN07P0001E22
FARO_FV263	cAMP-dependent transcription factor ATF-1	Sparus aurata	cDN07P0001M01
FARO_FV264	desert hedgehog	Sparus aurata	cDN07P0005M14
FARO_FV265	Ca(2+)-binding S-100 protein	Sparus aurata	cDN07P0006A18
FARO_FV266	COL1A3	Sparus aurata	cDN07P0006G06
FARO_FV267	SP1	Sparus aurata	cDN08P0003F23
FARO_FV268	TGFBI	Sparus aurata	cDN08P0003H13
FARO_FV269	fms-related tyrosine kinase 3 (FLT3)	Sparus aurata	cDN08P0006O08
FARO_FV270	LAMININ C1	Sparus aurata	cDN09P0001H20
FARO_FV271	Tetraodon full-lenght cDNA	Sparus aurata	cDN09P0003A09
FARO_FV272	Thrombospondin 1	Sparus aurata	cDN09P0004K06
FARO_FV273	ACVR1 activin A receptor	Sparus aurata	cDN09P0005L12
FARO_FV274	Osteonectin	Sparus aurata	cDN09P0006G12
FARO_FV275	receptor tyrosine kinase Kdr (kdr)	Sparus aurata	cDN09P0006H21

FARO_FV276	STAT3	Sparus aurata	cDN10P0001H24
FARO_FV277	COL2A1/COL5A2	Sparus aurata	cDN10P0002C17
FARO_FV278	calmodulin 1b	Sparus aurata	cDN10P0005E01
FARO_FV279	matrix Gla protein (MGP)	Sparus aurata	cDN10P0005G18
FARO_FV280	phospholipase A2	Sparus aurata	cDN11P0001G04
FARO_FV281	COL1A3/COL1A1	Sparus aurata	cDN11P0002E17
FARO_FV282	COL1A3/COL1A1	Sparus aurata	cDN11P0002G12
FARO_FV283	COL12A1	Sparus aurata	cDN11P0003A07
FARO_FV284	fibroblast growth factor receptor drosophila	Sparus aurata	cDN11P0003E19
FARO_FV285	retinoblastoma binding protein 6-like (rbbp6l)	Sparus aurata	cDN11P0004M01
FARO_FV286	FIBRONECTIN1	Sparus aurata	cDN11P0004M02
FARO_FV287	COL1A1	Sparus aurata	cDN11P0004P07
FARO_FV288	Alkaline phosphatase	Sparus aurata	cDN11P0005A21
FARO_FV289	COLV/XIA1	Sparus aurata	cDN11P0005F07
FARO_FV290	COL2A1/COL5A2	Sparus aurata	cDN12P0001E12
FARO_FV291	Sox6a (Sox6a)	Sparus aurata	cDN12P0002I17
FARO_FV292	retinoblastoma binding protein 6	Sparus aurata	cDN12P0002O16
FARO_FV293	insulin-like growth factor I receptor form A	Sparus aurata	cDN12P0003G16
FARO_FV294	thrombospondin 1 (thbs1)	Sparus aurata	cDN12P0004D19
FARO_FV295	receptor tyrosine kinase Kdr	Sparus aurata	cDN12P0004D23
FARO_FV296	fibronectin 1	Sparus aurata	cDN12P0004H03
FARO_FV297	retinoblastoma binding protein 6-like (rbbp6l)	Sparus aurata	cDN12P0004H04
FARO_FV298	RAMP3 mRNA for receptor activity modifying	Sparus aurata	cDN12P0005H19
FARO_FV299	cAMP-dependent transcription factor ATF-1	Sparus aurata	cDN12P0006L07
FARO_FV300	tenascin-C	Sparus aurata	cDN13P0001C23
FARO_FV301	calmodulin	Sparus aurata	cDN13P0003B12
FARO_FV302	Thrombospondin 4	Sparus aurata	cDN13P0003D07
FARO_FV303	ets variant gene 6 protein (ETV6)	Sparus aurata	cDN13P0003I05
FARO_FV304	thrombospondin 4	Sparus aurata	cDN13P0004C23
FARO_FV305	biglycan	Sparus aurata	cDN14P0001E13
FARO_FV306	LTBP-1	Sparus aurata	cDN14P0001L22
FARO_FV307	COL12A1	Sparus aurata	cDN14P0001N22
FARO_FV308	Thrombospondin 3/4	Sparus aurata	cDN14P0003C14
FARO_FV309	Calmodulin1	Sea bass	cDN22P0002B04
FARO_FV310	Matrix gla protein	Sea bass	cDN22P0002C23
FARO_FV311	TRAFfamily member	Sea bass	cDN22P0002F11
FARO_FV312	TIMP-2b	Sea bass	cDN22P0003C19
FARO_FV313	Matrix gla protein	Sea bass	cDN22P0004D20
FARO_FV314	Stat3	Sea bass	cDN22P0004L18
FARO_FV315	vitronectin	Sea bass	cDN22P0004M08
FARO_FV316	Calcium binding protein P22	Sea bass	cDN22P0005C08
FARO_FV317	Calmodulin1	Sea bass	cDN22P0006A11
FARO_FV318	Kruppel-like factor 10 TFGIF	Sea bass	cDN22P0006B08
FARO_FV319	Tetraodon full-lenght cDNA	Sea bass	cDN22P0006O21
FARO_FV320	Proto-oncogene protein c-Fos	Sea bass	cDN23P0004C19
FARO_FV321	Calmodulin1	Sea bass	cDN24P0002I18

FARO_FV322	Proto-oncogene protein c-Fos	Sea bass	cDN24P0002L04
FARO_FV323	TRAFfamily member	Sea bass	cDN24P0003A13
FARO_FV324	Matrilin 1	Sea bass	cDN25P0002M24
FARO_FV325	Matrix gla protein	Sea bass	cDN25P0003A22
FARO_FV326	Sox9	Sea bass	cDN25P0004A18
FARO_FV327	TF Pu.1	Sea bass	cDN25P0004F03
FARO_FV328	Epididymal sperm binding protein!!	Sea bass	cDN25P0005E19
FARO_FV329	Osteonectin	Sea bass	cDN25P0006J04
FARO_FV330	Sox3	Sea bass	cDN26P0001O19
FARO_FV331	Sox1a (fugu)	Sea bass	cDN26P0003F04
FARO_FV332	COL1A1	Sea bass	cDN26P0003K14
FARO_FV333	Sox14a (fugu)	Sea bass	cDN26P0004A11
FARO_FV334	Fibronectin 1	Sea bass	cDN27P0003N14
FARO_FV335	Laminin C1	Sea bass	cDN27P0004J15
FARO_FV336	Laminin alpha 4	Sea bass	cDN27P0005B18
FARO_FV337	Calcium binding protein P22	Sea bass	cDN28P0001B19
FARO_FV338	disintegrin-like and metalloprotease with thron	Sea bass	cDN28P0001P15
FARO_FV339	leptin	Sea bass	cDN28P0002F13
FARO_FV340	Caveolin-1	Sea bass	cDN28P0002J24
FARO_FV341	PTHrP	Sea bass	cDN28P0003M07
FARO_FV342	fibronectin 1a	Sea bass	cDN28P0004B07
FARO_FV343	sox 17	Sea bass	cDN28P0005N10
FARO_FV344	S100 calcium binding protein	Sea bass	cDN28P0006O07
FARO_FV345	ADAM-TS2	Sea bass	cDN28P0007I02
FARO_FV346	pontin	Sea bass	cDN29P0005I14
FARO_FV347	COL5A2/Col2A2	Sea bass	cDN30P0001L18
FARO_FV348	SOX8	Sea bass	cDN30P0004I02
FARO_FV349	PU.1	Sea bass	cDN30P0005I05
FARO_FV350	S100 calcium binding protein	Sea bass	cDN31P0006J07
FARO_FV351	COL1A1	Sea bass	cDN32P0001L07
FARO_FV352	S100 calcium binding protei	Sea bass	cDN32P0003A05
FARO_FV353	acid phosphatase 5, tartrate resistant (acp5)	Sea bass	cDN32P0003H01
FARO_FV354	TRAF family member-associated NF-kB activato	Sea bass	cDN32P0005A05
FARO_FV355	ets variant gene 6 protein (ETV6)	Sea bass	cDN35P0002J06
FARO_FV356	Kallmann syndrome 1	Sea bass	cDN35P0004O21
FARO_FV357	Annexin 2a	Sparus aurata	SAPD12001
FARO_FV358	Annexin a11	Sparus aurata	SAPD22824
FARO_FV359	Discs, large homolog 7	Sparus aurata	SAPD04105
FARO_FV360	Cyclin- dependent kinase inhibitor 3	Sparus aurata	SAPD22138
FARO_FV361	EF-hand domain-containing protein 2	Sparus aurata	SAPD23800
