# Supplementary material for: Insights into shell deposition in the Antarctic bivalve Laternula elliptica: gene discovery in the mantle transcriptome using 454 pyrosequencing
Source: BMC Genomics. 2010 Jun 8;11:362. doi: 10.1186/1471-2164-11-362 (PMC2896379; doi:10.1186/1471-2164-11-362)
Supplement: Additional file 2 — Table S2. List of genes and accession numbers comprising in-house database of the family 2 GPCRs. [file 1471-2164-11-362-S2.PDF]

Code	*gene origin*	*species*	*designation*	*description*
Faro_JC_01	110349437	sea bream	PCR product	VIP
Faro_JC_02	110349435	sea bream	PCR product	PACAP
Faro_JC_03	57283057	sea bream	cDNA clone	PAC1bR
Faro_JC_04	57283055	sea bream	cDNA clone	PAC1aR
Faro_JC_05	48843195	sea bream	cDNA clone	VPAC2
Faro_JC_06	125838206	sea bream	cDNA clone	GIPR-like
Faro_JC_07	50841441	Drosophila	mRNA	PDFR-pigment dispersing factor receptor
Faro_JC_08	22026873	Drosophila	mRNA	family 2 GPCR
Faro_JC_09	161076587	Drosophila	mRNA	family 2 GPCR
Faro_JC_10	24653338	Drosophila	mRNA	family 2 GPCR
Faro_JC_11	161077788	Drosophila	mRNA	family 2 GPCR
Faro_JC_12	71981663	C.elegans	mRNA	PDFR-pigment dispersing factor receptor
Faro_JC_13	193211300	C.elegans	mRNA	family 2 GPCR
Faro_JC_14	71983793	C.elegans	mRNA	family 2 GPCR
Faro_JC_15	609351	Acheta domesticus	mRNA	Dhreceptor
Faro_JC_16	437284	Manduca sexta	mRNA	Dhreceptor
Faro_JC_17		zebrafish	PCR product	PACAPA
Faro_JC_18		zebrafish	PCR product	PACAPB
Faro_JC_19	163954959	Takifugu	Gene	PACAPB
Faro_JC_20	163954957	Takifugu	Gene	PACAPB
Faro_JC_21	publication	Tunicate	Gene	pacap1
Faro_JC_22	publication	Tunicate	Gene	pacap2
Faro_JC_23	153266792	Human	Gene	PACAP
Faro_JC_24	148706717	Mouse	Gene	PACAP
Faro_JC_25	Ensembl	Frog	Gene	PACAP
Faro_JC_26	Ensembl	Chicken	Gene	PACAP
Faro_JC_27	4507897	Human	Gene	VIP
Faro_JC_28	Ensembl	Chicken	Gene	VIP
Faro_JC_29	148671621	Mouse	Gene	VIP
Faro_JC_30	11345450	Human	Gene	Secretin
Faro_JC_31	publication	Goldfish	mRNA	VIP

Faro_JC_32	publication	Goldfish	mRNA	VIP
Faro_JC_33	163954955	Takifugu	Gene	VIP
Faro_JC_34	Ensembl	Xenopus	Gene	VIP
Faro_JC_35	11034841	Human	Gene	GHRH
Faro_JC_36	Ensembl	Chicken	Gene	GHRH
Faro_JC_37	76880474	Human	mRNA	CALC
Faro_JC_38	20302161	Human	mRNA	GCG
Faro_JC_39	39995098	Human	mRNA	PTH
Faro_JC_40	30218	Human	mRNA	CRFbp
Faro_JC_41	Ensembl	Medaka	mRNA	VIP
Faro_JC_42	161878880	zebrafish	mRNA	VIP
Faro_JC_43		Trout	mRNA	VIP
Faro_JC_44	38175225	Halocynthia roretzi	PCR product	PACAP-like
Faro_JC_45		Sepioteuthis lessoniana	PCR product	PACAP-like
Faro_JC_46	38175237	Eriocheir japonica	PCR product	PACAP-like
Faro_JC_47	23978570	Periplaneta americana	PCR product	PACAP-like
Faro_JC_48	23978562	Hydra magnipapillata	PCR product	PACAP-like
Faro_JC_49	23978558	Dugesia japonica	PCR product	PACAP-like
Faro_JC_50	6456567	Petromyzon marinus	mRNA	GCG1
Faro_JC_51	10181174	Mouse	mRNA	PTH
Faro_JC_52	50979128	Canis familiaris	mRNA	PTH
Faro_JC_53	47086141	zebrafish	mRNA	PTH2
Faro_JC_54	4506267	Human	mRNA	PTH
Faro_JC_55	Ensembl	Chicken	mRNA	PTH
Faro_JC_56	Ensembl	Xenopus	mRNA	PTH
Faro_JC_57	47086141	zebrafish	mRNA	PTH1
Faro_JC_58	84309995	Takifugu	mRNA	PTH1
Faro_JC_59	34499964	Takifugu	mRNA	PTH2
Faro_JC_60	39995091	Human	mRNA	PTHrP
Faro_JC_61	Ensembl	Chicken	mRNA	PTHrP
Faro_JC_62	Ensembl	Xenopus	mRNA	PTHrP
Faro_JC_63	39995091	zebrafish	mRNA	PTHrPB
Faro_JC_64	112807240	zebrafish	mRNA	PTHrPA

Faro_JC_65		8546805	Takifugu	mRNA	PTHrPA
Faro_JC_66		84309993	Takifugu	mRNA	PTHrPB
Faro_JC_67	Ensembl		Xenopus	mRNA	PTH-L
Faro_JC_68	Ensembl		Chicken	mRNA	PTH-L
Faro_JC_69	Ensembl		zebrafish	mRNA	PTH-L
Faro_JC_70		84309999	Takifugu	mRNA	PTH-L
Faro_JC_71		34592154	zebrafish	mRNA	TIP39
Faro_JC_72		119572865	Human	mRNA	TIP39
Faro_JC_73		148690870	Mouse	mRNA	TIP39
Faro_JC_74	Ensembl		Xenopus	mRNA	GCG
Faro_JC_75		56693261	zebrafish	mRNA	GCGA
Faro_JC_76		185136061	Trout	mRNA	GCGII
Faro_JC_77	Ensembl		Lamprey	mRNA	PRP
Faro_JC_78	Ensembl		Lamprey	mRNA	PACAP
Faro_JC_79	Ensembl		Lamprey	mRNA	VIP
Faro_JC_80	Ensembl		Lamprey	mRNA	PRP
Faro_JC_81	JGI		Amphioxus	Gene	family 2 GPCR
Faro_JC_82	JGI		Amphioxus	Gene	family 2 GPCR
Faro_JC_83	JGI		Amphioxus	Gene	family 2 GPCR
Faro_JC_84	JGI		Amphioxus	Gene	family 2 GPCR
Faro_JC_85	JGI		Amphioxus	Gene	family 2 GPCR
Faro_JC_86	JGI		Amphioxus	Gene	family 2 GPCR
Faro_JC_87	JGI		Amphioxus	Gene	family 2 GPCR
Faro_JC_88	JGI		Amphioxus	Gene	family 2 GPCR
Faro_JC_89	JGI		Amphioxus	Gene	family 2 GPCR
Faro_JC_90		118790870	Anopheles gambiae	Gene	family 2 GPCR
Faro_JC_91		157124932	Aedes aegypti	Gene	family 2 GPCR
Faro_JC_92		170058376	Culex quinquefasciatus	Gene	family 2 GPCR
Faro_JC_93		120538157	zebrafish	Gene	CALR
Faro_JC_94		662795	Human	Gene	SCTR
Faro_JC_95		157412285	Takifugu	Gene	PAC1aR
Faro_JC_96		397520	Mouse	mRNA	PAC1
Faro_JC_97	Ensembl		lamprey	Gene	GLPR
Faro_JC_98		158294220	Anopheles gambiae	Gene	CRF-like
Faro_JC_99	Ensembl		Lamprey	Gene	family 2 GPCR

Faro_JC_100	161137861	Aedes aegypti	Gene	family 2 GPCR
Faro_JC_101	158291895	Anopheles gambiae	Gene	family 2 GPCR
Faro_JC_102	198421409	Ciona	gene	CRFR-like
Faro_JC_103	198429270	Ciona	Gene	CALR-like
Faro_JC_104	198432253	Ciona	Gene	CRFR-like
Faro_JC_105	198437590	Ciona	Gene	GCGR-like
Faro_JC_106	198429270	Ciona	Gene	CALR-like
Faro_JC_107	198415413	Ciona	Gene	PTHR-like
Faro_JC_108	198430700	Ciona	Gene	GCGR-like
Faro_JC_109	198437592	Ciona	Gene	GCGR-like
Faro_JC_110	198419503	Ciona	Gene	PTHR-like
Faro_JC_111	179879	Human	mRNA	CALCR
Faro_JC_112	158259456	Human	mRNA	CGRPR
Faro_JC_113	223717970	Human	mRNA	CRFR1
Faro_JC_114	38349112	Human	mRNA	CRFR2
Faro_JC_115	45545425	Human	mRNA	GIPR
Faro_JC_116	85567207	Human	mRNA	GLP1
Faro_JC_117	66365835	Human	mRNA	GLP2
Faro_JC_118	58530850	Human	mRNA	GHRH
Faro_JC_119	39995096	Human	mRNA	PTHR1
Faro_JC_120	39995097	Human	mRNA	PTHR2
Faro_JC_121	190340160	Danio	mRNA	PTHR1
Faro_JC_122	190339357	Danio	mRNA	PTHR2
Faro_JC_123	18859290	Danio	mRNA	PTHR3
Faro_JC_124	34484349	Human	mRNA	PAC1R
Faro_JC_125	38609718	Human	mRNA	SCTR
Faro_JC_126	196115237	Human	mRNA	VPAC1
Faro_JC_127	30583074	Human	mRNA	VPAC2
Faro_JC_128	58761178	Goldfish	mRNA	GLPR1
Faro_JC_129	4501944	Human	mRNA	ADM
Faro_JC_130	76559785	Takifugu	mRNA	CALR
Faro_JC_131	76559789	Takifugu	mRNA	CALR3
Faro_JC_132	84993707	Takifugu	mRNA	CALR
Faro_JC_133	257792887	Carp	mRNA	CRF
Faro_JC_134	46406039	Goldfish	mRNA	GCGR
Faro_JC_135	119371495	Takifugu	mRNA	CALR2

Faro_JC_136	125838205	Danio	mRNA	GLPR2
Faro_JC_137	157412283	Takifugu	mRNA	PAC1aR
Faro_JC_138	157412285	Takifugu	mRNA	PAC1bR
Faro_JC_139	169259763	Danio	mRNA	VPAC2
Faro_JC_140	190339641	Danio	mRNA	VPAC1
Faro_JC_141	197085598	Danio	mRNA	GHRHR
Faro_JC_142	257792885	Carp	mRNA	CRFR
Faro_JC_143	Ensembl	Lamprey	gene	CRFR
Faro_JC_144	Ensembl	Lamprey	Gene	family 2 GPCR
Faro_JC_145	cDN19P0001A09	Oyster	mRNA	family 2 GPCR
Faro_JC_146	OstraBG1	Oyster Ang	PCR product	family 2 GPCR
Faro_JC_147	OstraCMI	Oyster Ang	PCR product	family 2 GPCR
Faro_JC_148	*cDN37P0004G16*	Oyster	mRNA	family 2 GPCR
Faro_JC_149	*cDN19P0003B14*	Oyster	mRNA	family 2 GPCR
Faro_JC_150	*cDN19P0001A09*	Oyster	mRNA	family 2 GPCR
Faro_JC_151	cDN18P0003E06	Mollusc	mRNA	family 2 GPCR
Faro_JC_152	37496574	Oyster	mRNA	CALR
Faro_JC_153	157688306	Goldfish	mRNA	AMY
Faro_JC_154	109255169	Human	mRNA	AMY
Faro_JC_155	225706817	Osmerus	mRNA	AMY
Faro_JC_156	157756892	Cbiggsae	gene	AMY-like
Faro_JC_157	193206090	Celegans	gene	AMY-like
Faro_JC_158	27754168	Mouse	mRNA	NPY
Faro_JC_159	5326817	Drosophila	mRNA	NPY-like
Faro_JC_160	24644522	Drosophila	mRNA	NPYR
Faro_JC_161	118783455	Aenophles	Gene	NPYR
Faro_JC_162	6580600	Lymnaea	mRNA	NPY
Faro_JC_163	2173303	Human	mRNA	OxytocinR
Faro_JC_164	189095260	Human	mRNA	Vasopressin
Faro_JC_165	33149326	Mouse	mRNA	VasopressinR
Faro_JC_166	6981315	Mouse	mRNA	Oxytocin
Faro_JC_167	92121714	Octopus	mRNA	Oxytocin
Faro_JC_168	13516973	Aplysia	mRNA	Conopressin
Faro_JC_169	387928	Drosophila	DNA	ADK
Faro_JC_170	55140542	Periplaneta americana	mRNA	ADK
Faro_JC_171	1085062	Locusta migratoria	mRNA	ADKII

Faro_JC_172	1085060	Locusta migratoria	mRNA	ADKI
Faro_JC_173	1008122	Locusta migratoria	mRNA	ADKIII
Faro_JC_174	6978506	Mouse	mRNA	ADM
Faro_JC_175	24650317	Drosophila	mRNA	PDF
Faro_JC_176	112982891	Bombyx mori	mRNA	PDF
